# Effect of grazing and climatic factors on biodiversity-ecosystem functioning relationships in grassland ecosystems - a case study of typical steppe in Inner Mongolia, China

**DOI:** 10.3389/fpls.2023.1297061

**Published:** 2023-12-22

**Authors:** Yiran Zhang, Wuriliga, Pengtao Liu, Ruyue Fan, Jing Guo, Li Liu, Yong Ding

**Affiliations:** ^1^ Inner Mongolia Key Laboratory of Grassland Conservation Ecology, Grassland Research Institute, Chinese Academy of Agricultural Sciences, Hohhot, China; ^2^ Graduate School of Chinese Academy of Agricultural Sciences, Beijing, China; ^3^ Inner Mongolia Ecology and Agrometeorology Centre, Hohhot, China

**Keywords:** Inner Mongolia steppe, grazing intensity, biodiversity, aboveground productivity, climatic fluctuation

## Abstract

Biodiversity underpins grassland ecological functions and productive capacities. By studying the mechanisms for the maintenance of species diversity in animal communities, we can provide important theoretical guidance for the optimization of grazing management and biodiversity protection. The typical grassland of Xilingol in Inner Mongolia, China, was used as the experimental area, and a grazing intensity experiment was set up. This consisted of four gradient levels that were grazed by sheep, which were available for continuous monitoring, namely control standard sheep unit·day·hectare^-1^·year^-1^ (CK, 0 SSU·d·hm^-2^y^-1^), light grazing (LG, 170 SSU·d·hm^-2^·y^-1^), moderate grazing (MG, 340 SSU·d·hm^-2^·y^-1^), and high grazing (HG, 510 SSU·d·hm^-2^·y^-1^). Nine consecutive years of multi-indicator monitoring of vegetation was carried out from 2014–2022, using monitoring data coupled with time series and inter-annual climatic (relative moisture index, RMI) fluctuations. This was done to analyze the impacts of disturbances, such as grazing use and climatic fluctuations, on the diversity of species and above-ground productivity of the community, thereby exploring the relationship between diversity and productivity, and provide possible explanations for the emergence of a range of ecological responses. The statistical analysis methods used were One-way Analysis of Variance (ANOVA), general linear regression and mixed-effects models. The main conclusions of this study are as follows: (1) The grassland in the experimental area under CK had the highest diversity and productivity and the ecosystem was better able to buffer the negative impacts of climatic drought. Furthermore, the effect of climate on productivity and diversity weakened as the intensity of grazing increased. (2) LG to MG had a constant diversity. (3) Grazing utilization changed the relationship between community species diversity and aboveground productivity by releasing spatial community resources, altering the structure of plant communities, weakening competitive exclusion, and strengthening complementary effects. However, under all of the conditions there is a brief stage in the time series when diversity is stimulated to increase, and the higher the grazing intensity, the earlier this occurs.

## 1 Introduction

In recent years, the increasing threat to the biodiversity of various ecosystems due to the impacts of climate change and human activities has resulted in the rapid disappearance of many fragile ecosystems (Alverson et al., 2003; Seddon et al., 2016; Meng et al., 2021). Consequently, research on biodiversity conservation has become a crucial topic in the fields of ecological and environmental sciences (Hooper et al., 2005; Vellend et al., 2017). Grasslands are important ecosystems as they cover approximately 5.25 billion hectares (40.5%) of the world’s land area, except for Greenland and Antarctica (China Green Times, 2018). Grasslands comprise of two primary categories: tropical (savannas) and temperate grasslands. These areas are primarily located in the intermediate zone between forests and deserts. Most of the world’s grasslands play key ecological and production roles, sustain livestock development, and safeguard ecosystem functions in dry and semi-arid areas (Suttie et al., 2005; Wu et al., 2015). They are commonly referred to as “meat, milk, carbon, and money banks.”

However, the ecological foundation of these grasslands is complex. Climate change, such as warming and alterations in precipitation patterns, as well as human intervention (e.g., overgrazing), has dramatically transformed the structure and species diversity of grassland ecosystems and their functions (Hickman et al., 2004; Heisler-White et al., 2008; Harrison et al., 2015; Ivits et al., 2016; Li et al., 2016; Ma et al., 2017). Consequently, grassland degradation, plant diversity loss, and ecosystem function decay have threatened global ecological security and the growth of agriculture and animal husbandry industries considerably (Nellemann and Corcoran, 2010; An et al., 2019). Therefore, maintaining and improving the ecological function and productivity of grasslands, with biodiversity as a representative indicator, has become a crucial theoretical and practical requirement for addressing global climate change and preventing land desertification.

In recent years, a series of important research advances have been made on grassland biodiversity maintenance mechanisms and the relationship between biodiversity and ecosystem functioning, especially productivity. These advances are of great value in guiding the development of more multidimensional and in-depth research, as well as grassland biodiversity conservation, restoration, and reorganization. The intermediate disturbance hypothesis and grazing optimization theory proposed by numerous scholars have had the most profound impacts on the study and conservation of grassland biodiversity and productivity. The intermediate disturbance hypothesis suggests that grassland ecosystems have a certain ability to regulate external disturbances, and that the intermediate disturbance frequency can maintain high species diversity and productivity. However, it has also been suggested that the intermediate disturbance hypothesis is not valid for arid grasslands with harsh environmental conditions (Sasaki et al., 2008). Throughout the large number of research cases, some follow the intermediate disturbance hypothesis, whereas some do not, and some have mixed responses, which suggests that the intermediate disturbance hypothesis still has large limitations. Therefore, further research is required that takes into consideration different ecosystems. The grazing optimization theory suggests that herbivores and forage evolve synergistically and that rational grazing is beneficial to grassland vegetation. The main theoretical basis of the grazing optimization theory is plant compensatory growth, which is the phenomenon in which plants are adapted to herbivore harvesting disturbances through the regulation of phenotypic quantitative traits and physiology. These adaptations include the increase in the number of divisions due to the breaking of the apical dominance, an increase in the rate of leaf photosynthesis, and a redistribution of storage resources (Yuan, 2019). However, the grazing optimization hypothesis is not necessarily true and is closely related to the status of plants before and after feeding and environmental conditions, as well as to different grassland types, grazing utilization methods, and plant species (Holechek, 1981; Hart and Balla, 1982). There are also studies on grasslands that do not provide sufficient evidence of grazing optimization (Gong et al., 2015). Grasslands, as ecosystems with a production function, must focus on their formation, maintenance, and responses to grazing disturbances and climate change in terms of productivity. Biodiversity is the main process parameter for studying changes in disturbed subjects resulting from disturbance factors, making it crucial to investigate the relationship between biodiversity and productivity. It has been argued that selection and complementary effects explain the principle of increased diversity to increased productivity (Grime, 1998; Loreau, 2000; Fu et al., 2014; Mensah et al., 2016), whereas the competitive exclusion theory suggests that in eutrophic environments, an increase in productivity reduces diversity. Overall, the relationship between diversity and productivity is complex, with positive, negative, uncorrelated, and nonlinear correlations.

To fully illustrate the effects of grazing on biodiversity in grassland grazing ecosystems and to determine how this change affects productivity, as well as to explore the mitigating and enhancing effects of inter-annual climate fluctuations, a gradient experiment on grazing intensity was set up in a typical grassland of the Xilinguole League, Inner Mongolia. The experiment ran from 2014 to 2022. This study was set out to illustrate the changes in grassland plant community structure, biodiversity and productivity and the intrinsic relationship between them under the changes of multiple elements such as grazing intensity, climate fluctuation, and time continuation by means of 9 years of successive observations, therefore providing a theoretical basis for the exploration of a sustainable grazing management system, and also for the exploration of the biological relationship between biodiversity and productivity.

## 2 Materials and methods

### 2.1 Study area

**Figure 1:**
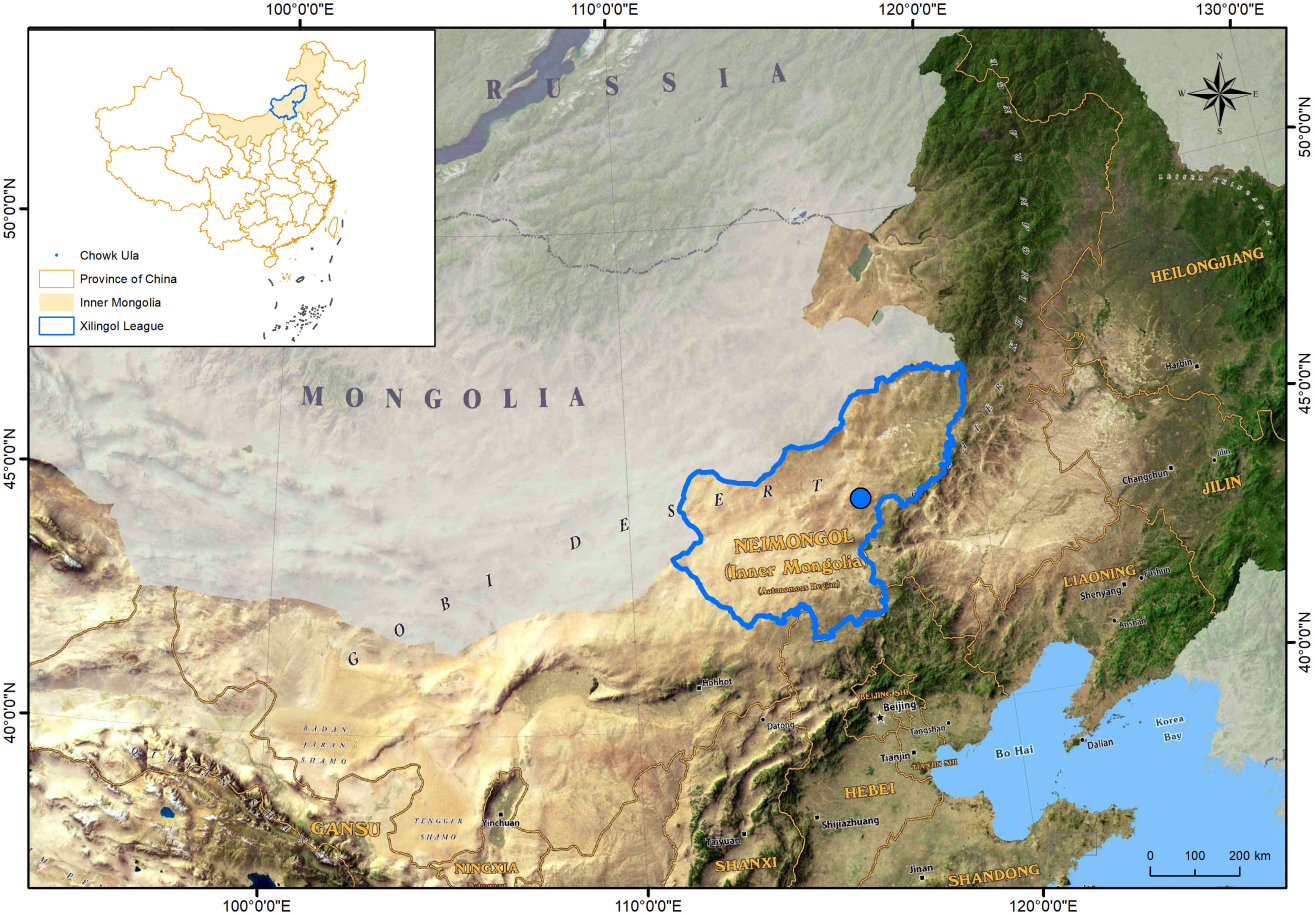
Geographical location of test area.

**Figure 2:**
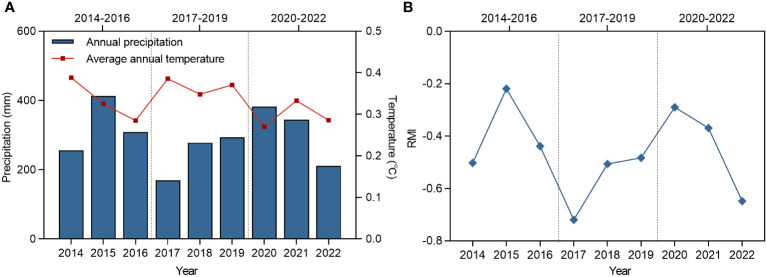
The changes of annual average temperature, annual precipitation and RMI in Xilinhot from 2014 to 2022. **(A)** Changes in mean annual temperature and annual precipitation in Xilinhot, 2014-2022. **(B)** Changes in RMI in Xilinhot, 2014-2022. RMI represents relative moisture index.

The study site is located in Chowk Ula, Xilinhot, Inner Mongolia, China (44°15′24.43″N ~ 44°15′40.66″N, 116°32′08.16″E ~ 116°32′28.32″E), and with an elevation of approximately 1111 m–1121 m (**Figure 1**). The average annual temperature and precipitation of the study area is approximately 2.6°C and 283 mm (1953–2022), respectively, thereby categorizing this area to the temperate semi-arid grassland climate zone. The average annual temperature of the climate within the nine year study period (2014–2022) was 0.3°C, the highest temperature was recorded in 2014 (0.4°C) and the lowest was in 2020 (0.3°C). The average annual precipitation within this period was 295 mm, the maximum annual precipitation was 413 mm (2015), and the minimum annual precipitation was 169 mm (2017). Both indicators exhibited a downward trend. The annual precipitation, average temperature, and relative moisture index (RMI) were studied to determine the inter-annual fluctuation characteristics of the climate (**Figure 2**). The data were derived from monthly meteorological data for Xilinhot, Inner Mongolia.

The relative moisture index (M) is one of the indexes to characterize the balance between precipitation and evaporation in a certain period of time. The larger the index, the more humid it is. The calculation formula is as follows (General Administration of Quality Supervision, Inspection and Quarantine of the People's Republic of China, 2017):

The RMI is one of the indices used to characterize the balance between precipitation and evaporation in a certain period of time. The larger the index, the more humid the environment. The calculation formula is as follows (General Administration of Quality Supervision, Inspection and Quarantine of the People's Republic of China, 2017):


(1) $$\begin{array}{c} M=\frac{P-PE}{PE} \end{array}$$

where *M* is the RMI, *P* is the amount of precipitation in mm over a period of time, and *PE* is the possible evapotranspiration for a certain period of time in mm.


(2) $$\begin{array}{c} PE_{m}=16.0\times {\left(\;\frac{10T_{i}}{H}\;\right)}^{A} \end{array}$$

where *PE_m_* is the possible evapotranspiration, which is the monthly possible evapotranspiration in mm/month; *T_i_* is the monthly average temperature in °C; *H* is the annual heat index; *A* is a constant.

Calculation method of monthly heat index *H_i_*:


(3) $$\begin{array}{c} H_{i}={\left(\;\frac{T_{i}}{5}\;\right)}^{1.514} \end{array}$$

The calculation method of annual heat index *H*:


(4) $$\begin{array}{c} H=\sum_{i=1}^{12}H_{i}=\sum_{i=1}^{12}{\left(\;\frac{T_{i}}{5}\;\right)}^{1.514} \end{array}$$

The calculation method of constant *A*:


(5) $$\begin{array}{c} A=6.75\times 10^{-7}H^{3}-7.71\times 10^{-5}H^{2}+1.792\times 10^{-2}H+0.49 \end{array}$$

When the monthly average temperature *T_i_* ⩽ 0°C, the monthly heat index *H_i_* = 0, and the monthly possible evapotranspiration *PE_m_* = 0 (mm/month).

The grassland type in this area is a typical grassland, the soil is chestnut, and plant species are abundant. The life function groups were divided into four different plant functional groups (PFGS) (**Table 1**): perennial grasses (PG), perennial forbs (PF), shrubs and semi-shrubs (SS), and annuals and biennials (AB).

**Table 1:** Species table of the study area.

PFGS	Species	
PG	*Leymus chinensis*	*Stipa krylovii*
	*Cleistogenes squarrosa*	*Agropyron cristatum*
	*Achnatherum sibiricum*	*Koeleria macrantha*
PF	*Caragana korshinskii*	*Allium ramosum*
	*Allium anisopodium*	*Allium tenuissimum*
	*Astragalus galactites*	*Iris tenuifolia*
	*Melilotoides ruthenica*	*Aster altaicus*
	*Allium mongolicum*	*Carex dispalata*
	*Lappula myosotis*	*Dontostemon dentatus*
	*Potentilla tanacetifolia*	*Gueldenstaedtia verna*
	*Allium condensatum*	*Potentilla acaulis*
	*Erodium stephanianum*	*Sibbaldianthe bifurca*
	*Thalictrum aquilegiifolium var. sibiricum*	*Euphorbia esula*
	*Carex duriuscula*	*Astragalus scaberrimus*
	*Convolvulus ammannii*	*Chamaerhodos erecta*
	*Oxytropis myriophylla*	*Potentilla betonicifolia*
	*Allium bidentatum*	*Phlomis umbrosa*
SS	*Artemisia frigida*	*Lespedeza bicolor*
	*Philadelphus tenuifolius*	*Vincetoxicum sibiricum*
AB	*Chenopodium glaucum*	*Artemisia scoparia*
	*Dysphania aristata*	*Salsola collina*
	*Setaria viridis*	*Axyris amaranthoides*
	*Eragrostis pilosa*	*Euphorbia humifusa*
	*Chloris virgata*	*Artemisia sieversiana*
	*Chenopodium acuminatum*	*Silene aprica*
	*Lepidium apetalum*	

### 2.2 Experimental design

The test area was closed between 2007–2013 for rehabilitation; the grazing test platform was set up in 2014 and has been undergone grazing trials since then. Four grazing intensities (standard sheep unit·day·hectare^-1^·year^-1^, SSU·d·hm^-2^y^-1^), were set in the study area, including the control (CK, 0 SSU·d·hm^-2^y^-1^), light grazing (LG, 170 SSU·d·hm^-2^y^-1^), moderate grazing (MG, 340 SSU·d·hm^-2^y^-1^), heavy grazing (HG, 510 SSU·d·hm^-2^y^-1^). According to the initial grazing weight of 50 kg as a standard sheep unit conversion, the experimental animals were Ujimqin 2-year-old wethers. The grazing test area was 1.33 hm^2^ (after 2018, in order to enrich the content of the test layout, the second and fourth divisions were implemented in some grazing test areas). Grazing intensity treatments consisted of three replicates per space. Grazing in the plot began in mid-June every year and ended in mid-September.

### 2.3 Index acquisition and calculation

#### 2.3.1 Plant community index acquisition

From 2014 to 2022, in mid and late August of each year, five 1 m × 1 m quadrats were randomly set up in each plot of the grazing experimental area, and the species list was recorded in detail for each quadrat. Each plant in the quadrat was cut off, bagged according to species, taken back to the laboratory for deactivation at 105°C for 30 min and dried to a constant weight at 65°C to obtain the aboveground biomass index of each plant species, this was used as the basic data for the calculation of the diversity index.

Additionally, three 1.2 m × 1.2 m annual mobile enclosures were arranged in the LG, MG, and HG treatment plots, and 1 m × 1 m quadrats were set up in mid-September of the same year. The species list was recorded, and the plants in the quadrats were cut according to species. As per the test requirements, the plants were dried and weighed to obtain aboveground net primary productivity (ANPP) of the plant communities under different grazing intensities.

#### 2.3.2 Index calculation

The species diversity index in this study was selected from the commonly used Shannon-Wiener (H), Simpson (D), and Pielou (J) indices. The importance value (P) was expressed as the relative biomass of each species in the community (relative biomass = dry weight of a species in the community/total dry weight of all species in the community). The formulas for calculating each index is as follows:


(6) $$\begin{array}{c} H=-\sum P_{i}\times \log_{2}P_{i} \end{array}$$

(7) $$\begin{array}{c} D=1-\sum P_{\text{i}}^{\;2} \end{array}$$

where *P_i_* is the important value of the *i*th species.


(8) $$\begin{array}{c} J=\frac{H}{\ln S} \end{array}$$

where *H* is the Shannon-Wiener diversity index, and *S* is the number of species.

### 2.4 Statistical analyses

A One-way Analysis of Variance (ANOVA) was used to analyze changes in grassland plant community productivity under grazing use and climate fluctuations, ratio of ANPP of grassland plants under different grazing intensities to ANPP in 2014, changes in community species diversity indices over time, and community species diversity indices under different grazing intensities. General linear regression was used to analyze the response of grassland plant functional group productivity to climate fluctuations. Mixed-effects models were also used to analyze the relationships between the RMI and species diversity, species diversity and community productivity, and the RMI and community productivity under different grazing intensities.

One-way ANOVA and general linear regression analyses were performed using SPSS version 26.0 (IBM Corp., Armonk, NY, USA). Mixed-effects models were generated using the lme4 program package in R version 4.2.2. Graphing was performed using GraphPad Prism version 9.5.1 (GraphPad Software Company, San Diego, California, USA). The data were expressed as mean ± SE. Differences were considered statistically significant at *p*< 0.05.

## 3 Results

### 3.1 ANPP and structure of plant communities

#### 3.1.1 ANPP of plant communities

In terms of RMI (**Figure 2**), the mean value of the index for Stage II was lower than those of Stages I and III, and it was generally in a relatively dry stage. In Stage II, community and perennial plant ANPP remained stable in CK, LG, and MG, whereas in HG, perennial ANPP decreased significantly, and community ANPP increased significantly. In Stage III, community and perennial plant ANPP significantly increased in CK and LG compared to the previous two stages due to the increase in RMI, MG perennial plant ANPP increased compared to the previous two stages, and HG perennial plant ANPP only significantly increased compared to Stage II.

**Figure 3:**
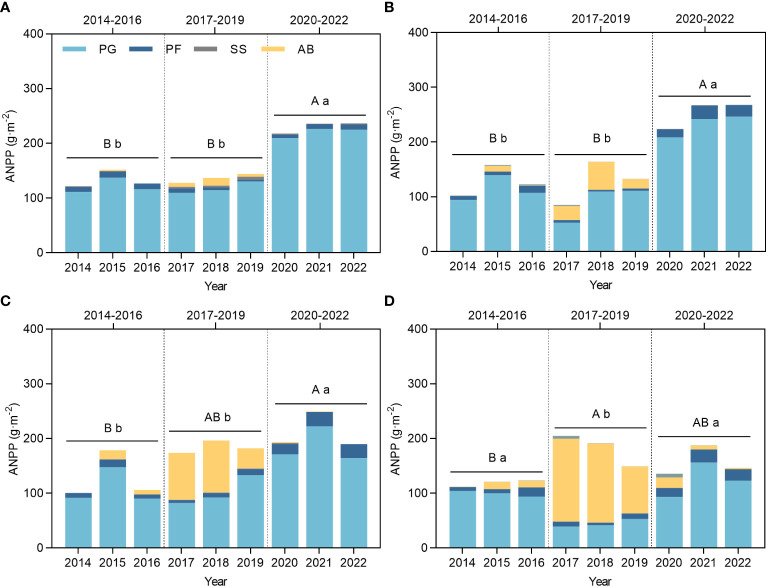
Changes in ANPP of grassland plants under grazing and climate fluctuations from 2014 to 2022. **(A)** ANPP of CK. **(B)** ANPP of LG. **(C)** ANPP of MG. **(D)** ANPP of HG. CK represents control standard, LG represents light grazing, MG represents moderate grazing, and HG represents heavy grazing. PG represents perennial grasses, PF represents perennial forbs, SS represents shrubs and semi-shrubs, and AB represents annuals and biennials. ANPP represents aboveground net primary productivity. Capital letters indicate significant differences in the mean ANPP of the plant community between Stage I, II, III. Lowercase letters indicate significant differences in the mean ANPP of the perennial plant community (PG, PF, SS) between Stage I, II, III. Different letters indicate that the difference is significant at the *p<* 0.05 level, the same below.

The grassland plant communities in the experimental area were subjected to various ecological effects resulting from the combined influence of climate (primarily rainfall or RMI), and environmental and anthropogenic disturbances. Grazing utilization intensities emerged as the dominant factor influencing these effects (**Figure 3**). Community productivity is a key metric for evaluating the responses to external factors, including disturbances. This study examined changes in ANPP by incorporating climatic fluctuations and the duration of grazing utilization across three stages: Stage I (2014–2016), Stage II (2017–2019), and Stage III (2020–2022). Community productivity results showed that the community constructed by accumulating species and the plant community were formed by removing AB and this showed no significant differences (*p* > 0.05) between Stages I and II, but both were significantly lower than Stage III in CK (**Figure 3A**) and LG (**Figure 3B**). While MG (**Figure 3C**) and HG (**Figure 3D**) increased dramatically owing to the productivity of AB in Stage II, the 3-year averages accounted for 105.53% and 119.28% of the average ANPP, which were much higher than those of CK and LG, as these amounted to 81.73% and 75.18%, respectively. Therefore, changes in the ANPP of the MG community compared to CK and LG were mainly characterized by no difference between Stage I, II, and III. While HG showed the highest ANPP in Stage II, which was significantly higher than that in stage I, and did not show any difference with stage III, the perennial plant ANPP in this stage was significantly lower than that in the other two stages, which were not significantly different (*p*< 0.05).

#### 3.1.2 Structure of plant community

Plant community structure was represented by the proportion of different functional groups in this study (**Supplementary Figure 1**). Taking the CK as a reference (**Supplementary Figure 1A**), the dynamic changes in CK can be attributed primarily to climate fluctuations. In CK, PG dominated the community, accounting for 83.8 to 96.0% of the total. Compared to Stage I, the percentages of AB and SS increased in the relatively dry Stage II, whereas PG and PF decreased. After the drought process of Stage II and the transition to relatively wet Stage III, the proportion of PG increased to greater than that of Stage I and the PF and SS decreased to a certain extent. Enhanced changes in some factors in LG (**Supplementary Figure 1B**), MG (**Supplementary Figure 1C**), and HG (**Supplementary Figure 1D**) compared with CK were considered to be related to grazing utilization and intensity. A large increase in the percentage of AB in the grazing-utilized grassland occurred in the second year of the trial (2015), reaching its highest percentage in 2017 and 2018. Both LG and MG almost entirely excluded AB from the community in 2020 and beyond, but a percentage remained in HG in 2020 and 2021. An increase in the proportion of AB in Stage II occurred at the expense of a reduction in PF and PG. Comparing Stage III with Stage I, most of the PF under the grazing treatments increased to some extent, showing a difference from CK, which may be the result of the ecological response to grazing utilization.

#### 3.1.3 Ecological response accumulation

**Figure 4:**
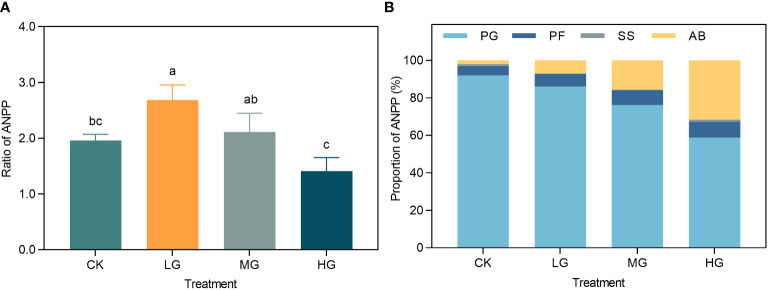
**(A)** Ratio of grassland plant ANPP to 2014 under different grazing intensities in 2020-2022. **(B)** Cumulative effects of grassland plant community structure under grazing utilization and climate fluctuations. CK represents control standard, LG represents light grazing, MG represents moderate grazing, and HG represents heavy grazing. PG represents perennial grasses, PF represents perennial forbs, SS represents shrubs and semi-shrubs, and AB represents annuals and biennials. ANPP represents aboveground net primary productivity.

We used three years of Stage III as replicated data, and analyzed the ratio of ANPP under different treatments to the initial year of the experiment (2014) as one of the cumulative characteristics of ecological response to grazing use (**Figure 4A**). Light grazing significantly increased the ANPP ratio compared to that of CK, but was not significantly different from that of MG and HG, therefore LG increased the ANPP. This analysis examined the proportion of the aggregate PFGS ANPP to the aggregate community ANPP for each treatment between 2014 and 2022, which served as a proxy for the cumulative impact of community composition (**Figure 4B**). The results indicated that as the grazing intensity increased and the percentage of PG ANPP decreased in the following order: CK > LG > MG > HG. Moreover, the PF and AB ANPP exhibited variable degrees of increase in correlation with grazing intensity, with a more significant increase in the proportion of AB.

### 3.2 Changes in species diversity

#### 3.2.1 Changes in the time series

**Figure 5:**
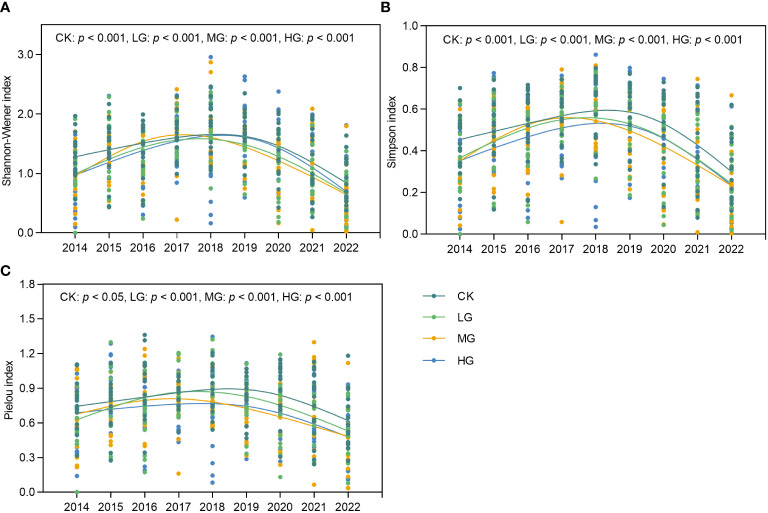
Changes in species diversity index of grassland communities over time from 2014 to 2022. **(A)** Shannon-Wiener index over time. **(B)** Simpson index over time. **(C)** Pielou index over time. CK represents control standard, LG represents light grazing, MG represents moderate grazing, and HG represents heavy grazing.

This study analyzed the temporal characteristics of the Simpson, Shannon-Wiener, and Pielou indices under different grazing treatments (**Figure 5**). Overall, the three diversity indices were significantly different (*p*< 0.05) under CK, LG, MG, and HG during the study period and showed a single-peaked curve characterized by peaks mostly occurring in Stage II, which was a relatively dry period. To illustrate the impact of grazing on diversity more clearly, we subtracted the diversity index calculated under the CK from that of the different grazing treatments and used the resulting comparative values to demonstrate the differential effects of grazing (**Supplementary Figure 2**). Over time, the three diversity indices exhibited a pattern of increase, followed by a decrease, compared with CK. The peaks of the Simpson and Shannon-Wiener indices for LG (**Supplementary Figures 2A,B**) occurred four years after the start of the trial, the Pielou index was in the third year (**Supplementary Figure 2C**), and all three indices were higher than that of CK in 2017 and 2018. The Simpson and Shannon-Wiener indices for MG (**Supplementary Figure 2D,E**) peaked three years after the commnecement of the experiment and were only higher than those of CK in the years of the respective peaks. Throughout the experiment, the Pielou index remained inferior to that of the CK (**Supplementary Figure 2F**). However, HG was significantly different (*p*< 0.05) from LG and MG (**Supplementary Figures 2G–I**). The HG peak appeared much earlier, with HG diversity being stimulated and exceeding that of CK in the second year of the trial, after which it was mostly lower than that of CK. In summary, our study found that grazing ecosystems were able to stimulate an increase in species diversity in the temporal dimension but sustained it for only 1–2 years, with this stimulatory effect occurring relatively earlier as grazing intensity increased.

#### 3.2.2 Cumulative effect of species diversity

**Figure 6:**
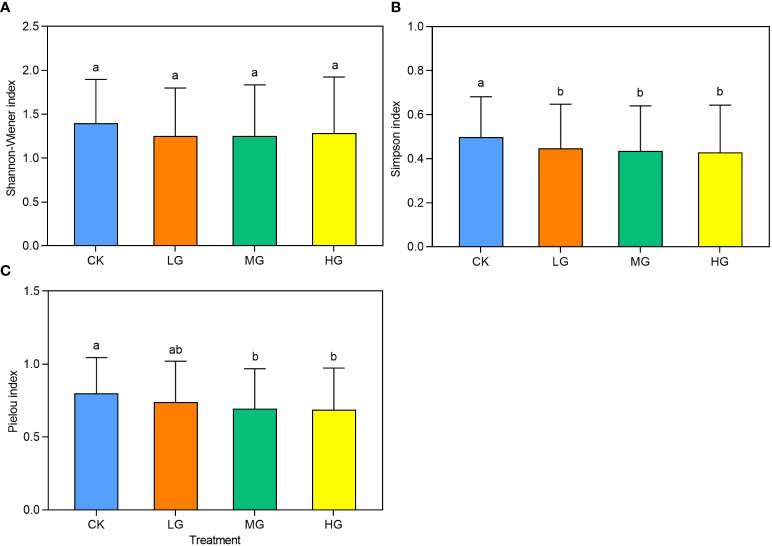
The community species diversity index under different grazing intensities from 2014 to 2022. CK represents control standard, LG represents light grazing, MG represents moderate grazing, and HG represents heavy grazing. **(A)** Shannon-Wiener index under different grazing intensities from 2014 to 2022. **(B)** Simpson index under different grazing intensities from 2014 to 2022. **(C)** Pielou index under different grazing intensities from 2014 to 2022.

The average diversity index values for each year across the different experimental treatments were used to indicate the overall impact on diversity (**Figure 6**). The analysis revealed that the Shannon-Wiener index did not vary significantly between grazing intensities. However, the Simpson and Pielou indices gradually decreased with increasing grazing intensity. The Simpson index indicated that CK had significantly higher values than LG, MG, and HG, whereas the Pielou index showed that CK had significantly higher values than MG and HG. However, CK was not significantly different from LG. Notably, there were no significant differences between CK and LG. The Simpson index captured the significance of differences between grazing intensity treatments better than the Shannon-Wiener and Pielou indices.

### 3.3 Impacts of climate fluctuations on species diversity and ANPP

#### 3.3.1 Response of major PFGS to climate fluctuations

**Figure 7:**
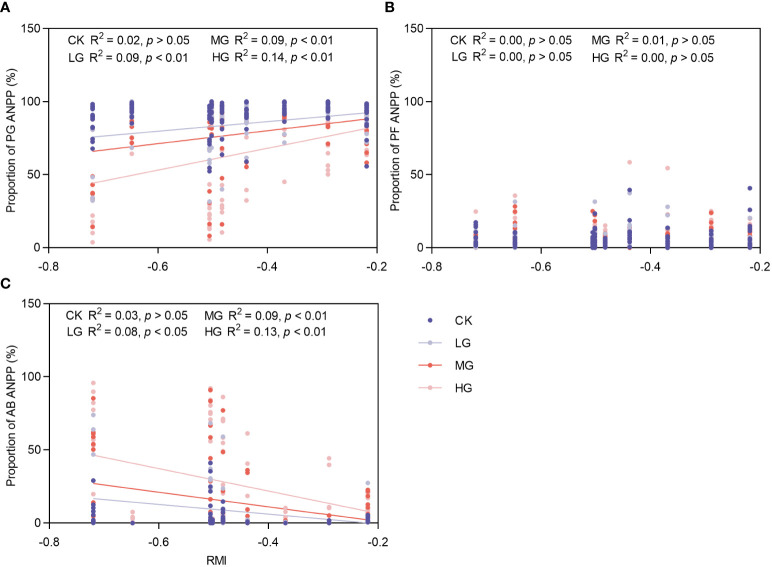
Response of changes in ANPP proportion of major functional groups to climatic fluctuations. CK represents control standard, LG represents light grazing, MG represents moderate grazing, and HG represents heavy grazing. PG represents perennial grasses, PF represents perennial forbs, SS represents shrubs and semi-shrubs, and AB represents annuals and biennials. ANPP represents aboveground net primary productivity. **(A)** Response of changes in ANPP proportion of PG to climatic fluctuations. **(B)** Response of changes in ANPP proportion of PF to climatic fluctuations. **(C)** Response of changes in ANPP proportion of AB to climatic fluctuations.

This study utilized the RMI to portray climate traits. Additionally, changes in the RMI were employed to illustrate inter-annual fluctuations in climate (**Figure 7**). Because of the disproportionately low ANPP of SS in the community, only the major PFGS were analyzed here. As the RMI increased (the degree of drought weakened), the ANPP of PG in LG, MG, and HG in the community increased significantly. The increase was greatest in HG, followed by MG, and smallest in LG, with no significant trend observed in CK. There was no significant response of PF ANPP to climatic fluctuations; AB ANPP showed significant decreases in LG, MG, and HG, with the largest decrease in HG, followed by MG, smallest decrease in LG, and no significant trend in CK.

#### 3.3.2 Species diversity in relation to ANPP of communities under climatic fluctuations

**Figure 8:**
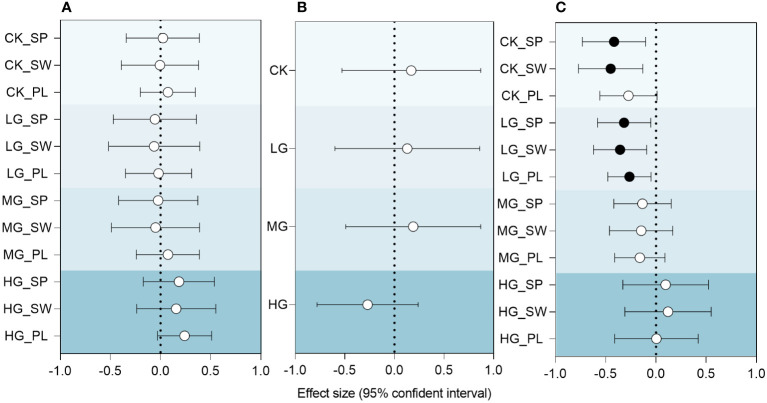
Relationships between RMI and species diversity index, species diversity index and ANPP of community, RMI and ANPP of community under different grazing intensities. **(A)** RMI and species diversity index. **(B)** RMI and ANPP of community. **(C)** Species diversity index and ANPP of community. SP: Simpson index; SW: Shannon-Wiener index; PL: Pielou index. CK represents control standard, LG represents light grazing, MG represents moderate grazing, and HG represents heavy grazing. RMI represents relative moisture index. Effect size is the standardized coefficient of a linear mixed-effects model estimated separately for each predictor variable. Solid circles indicate significant effects (*p*< 0.05), and hollow circles indicate nonsignificant effects.

From the results of the entire experimental timeline, the inter-annual differences in the RMI had different effects on plant community diversity under different treatments (**Figure 8A**). The results showed that the three diversity indices of CK were stable and did not show a trend in response to climatic drought and wetness. Relative to CK, LG and MG exhibited a negative response to increased relative wetness, while HG showed a positive response. This indicates that grazing utilization alters the trend tendency of community species diversity in response to climate change.

Community productivity in response to the relative humidity index (**Figure 8B**) was slightly lower in LG and slightly higher in MG than in CK, with CK, LG and MG showing positive trends, and HG showing negative trends. Although the relationship between the RMI and ANPP was not significant, the change in this tendency implied a significant change in the HG community, which may be related to the extensive growth of AB.

The correlation between species diversity index and community ANPP is shown in **Figure 8C**. This study revealed that grazing intensity alters the relationship between ANPP and species diversity indices. The control and LG species diversity indices were significantly negatively correlated with community productivity, whereas CK was not significantly correlated with MG and HG. Upon reaching HG, the relationship becomes insignificant, with a shift from negative to positive.

## 4 Discussion

### 4.1 Effects of grazing on species diversity of typical grassland plant communities in Inner Mongolia

Grazing utilization, especially intensive grazing, is a major factor altering the diversity and productivity of grassland ecosystems. When grazing is combined with climatic factors, the impact of the climate is often considered weaker (Collins and Barber, 1986; Zhao et al., 2003). Both the intermediate disturbance hypothesis and grazing optimization theory attempt to explain how rational grazing increases biodiversity and benefits ecosystems. The results of this study showed that LG and MG can increase or maintain relatively high ANPP, and LG can maintain species diversity as it was similar to CK, however there was no evidence suggesting that grazing would result in increased diversity. Additionally this finding aligns with previous studies as the predicted results of these studies confirm that species richness and diversity indices show a single-peak curve relationship with increasing grazing intensity, that is, plant diversity is highest at intermediate grazing intensity, and diversity decreases due to heavy grazing (Connell, 1978; Milchunas et al., 1988; Oba et al., 2001; Zhang et al., 2016; Joubert et al., 2017). However, research on the temporal dynamics of plant community diversity under grazing conditions is limited. Additionally, this study further demonstrated that grazing stimulates the ecosystem to increase biodiversity at a certain time compared to a non-grazing system, but this does not last long and then falls below the CK. This finding can be related to a 5-year grazing intensity-based experiment that confirmed that low loading rates favor sustainable grassland use and compensatory plant growth, and that the relationship between the compensatory growth of plant communities and loading rates can be modeled as a quadratic function with a downward opening or as a linear function with a negative slope, that is, as the loading rate increases, some systems exhibit undercompensatory growth, while others exhibit overcompensatory growth (Xue et al., 2010). The greater the grazing intensity, the earlier the excitation effect will appear, which may produce useful guidance for the optimization and adaptive management of grazing intensity over time. The priming effect on the time series under different grazing intensities was mainly related to changes in the community structure. In this study area, climate aridification caused a spatial release of the community, providing opportunities for AB to grow, which in turn appeared to increase the number and diversity of species in the community. However, from our experiments the addition of grazing and a disturbance factor, advanced and delayed the emergence of AB and increased the degree of dominance of AB in the community as the intensity of grazing increased. This result further confirms that the close relationship between grassland use patterns and species diversity depends on changes in species composition (Huo et al., 2014; Zhang et al., 2021).

### 4.2 Effects of climatic fluctuations on species diversity and ANPP of typical grassland plant communities in Inner Mongolia

Changes in precipitation and temperature are the primary indicators of global climate change and exert a substantial influence on both plant growth and diversity (Schultz and Halpert, 1995; Stanisci et al., 2005). In this study, we selected the RMI, which combines temperature and precipitation, to measure the level of dryness and wetness of the climate as the most scientific approach for analyzing this concept. However, our research findings revealed that climate did not have a substantial impact on the species diversity and ANPP of grazing ecosystem communities in this study. This can be attributed to factors such as the scale of the study and grazing utilization. At larger spatial scales, the gradient variation in climatic factors played a more prominent role than local-scale human activities, leading to a higher proportion of climate-related explanations for the distribution pattern of species diversity. However, at smaller spatial scales, the extent of the climate explanation has decreased (Bai et al., 2008; Fan et al., 2009). Furthermore, studies have shown that there is scale dependence in the relationship between the two, and that at large scales (e.g., regional or global scales), the relationship may be positive; however, at relatively small scales (e.g., local and landscape scales), there may be a one-peak function, a negative correlation, or no significant correlation between the two (Waide et al., 1999; Cornwell and Grubb, 2003; Ma et al., 2013; Hautier et al., 2018). This also reflects the difference between α- and β-diversity. The CK was the most stable ecosystem in terms of diversity, however this did not demonstrate any skewed trends toward changes in the RMI. This suggests that the spatial resources of the forbidden grassland were consistently occupied and utilized by PG. In addition, they display greater stability in arid environments and are less dependent on precipitation changes (Hoover et al., 2021). After grazing interference, the diversity index showed a negative relationship with the RMI, which was caused by the wet climate of the year. Perennial grasses are often nibbled to limit the occupation of its spatial resources, when the water resources are more adequate, its use of the functional group of the PG advantage, as well as the space left out of the grazing interference, through the competition for resources, further strengthens and expands the functional group of the PG to the exclusion of the AB. This results in a tendency to reduce diversity. When overgrazing occurs, a large amount of space in the community is completely freed, resources become redundant, and the onset of a wet climate conditions provide conditions for different plant species to survive, and provide an opportunity to increase the diversity of species in the community. Similarly, the wetter the climate, the higher the productivity of the non-heavily grazed utilization community, owing to the increase in the dominant PG. However, for heavily grazed communities, the increase in PG in wet years will exclude AB, but the increase in PG often does not complement or even overcompensate for the biomass of the excluded AB; thus, there is a tendency for the ANPP of the community to decline as the RMI increases.

### 4.3 Species diversity of plant communities in typical grassland grazing ecosystems in Inner Mongolia in relation to ANPP

The relationship between species diversity and productivity has been shown to take four main forms: positive (Tilman, 1997; Mittelbach et al., 2001), negative (Thompson et al., 2005), single-peaked (Kassen et al., 2000; Loreau, 2000), and uncorrelated (Grace et al., 2007]. The results of this study showed that in typical grassland grazing ecosystems, CK and LG ANPP were significantly negatively correlated with species diversity, which is in line with the theory of competitive exclusion. Although the CK ANPP was not at its maximum and was significantly lower than the LG ANPP, upon close examination of resource utilization, it appears that the CK space resources may be saturated. In CK, under the state of multi-year grazing ban, the ground was covered by a large amount of deadfall, spatial resources were crowed, light resources were restricted, PG was barely affected, and the growth of relatively low plants were somewhat restricted. Under LG conditions, freeing up a certain amount of space that tends to be occupied by more competitive PG, with plants such as PF being competitively crowded out. Although LG ANPP was the highest among the four grazing treatments, its diversity was not at its maximum, which is consistent with the theory of competitive exclusion and grazing optimization. This study also found that the relationship between species diversity and ANPP of the community was negatively tilted, but not significant, as grazing intensity increased to reach the MG, suggesting that competitive exclusion was attenuated under this grazing condition and that competitive exclusion and complementary effects may co-exist. Competitive exclusion plays a dominant role in relatively good climatic and dry years, and the compensatory effects of ABs increase community species diversity and further widen differences in functional traits. Plants utilize resources more efficiently, which in turn maintains diversity and contributes to increased productivity (Cavanaugh et al., 2014; Mensah et al., 2016; Pan et al., 2016). When the grazing intensity reaches HG, spatial resources are fully released, complementary effects are strengthened, and the relationship between productivity and diversity becomes positive.

## 5 Conclusion

In the typical grasslands of Inner Mongolia, forbidden grasslands have proven more effective in mitigating the adverse effects of climatic drought, with climate playing a lesser role in ANPP and diversity as grazing intensity increases. Light or moderate grazing maintains diversity but does not necessarily lead to an increase. There were brief periods within the time series when diversity was stimulated to increase, regardless of whether grazing was light, moderate, or heavy. Grazing modified the connection between the species diversity of the community and ANPP by releasing the spatial resources of the community, changing the structure of the plant community, weakening competitive exclusion, and strengthening complementary effects. The scope of this study was limited to small-scale and uncomplicated community structures in the research area that are prone to disruptions from grazing activities. Although several outcomes did not reach statistical significance, some trends offer valuable information. For instance, the impacts of both wet and dry climate changes on the diversity of grassland plant communities in the study region resulted in distinct trends.

## Data availability statement

The original contributions presented in the study are included in the article/**Supplementary Material**. Further inquiries can be directed to the corresponding authors.

## Author contributions

YZ: Conceptualization, Investigation, Software, Visualization, Writing – original draft. W: Software, Visualization, Writing – original draft. PL: Investigation, Writing – original draft. RF: Investigation, Writing – original draft. JG: Investigation, Writing – original draft. LL: Software, Visualization, Writing – review & editing. YD: Conceptualization, Funding acquisition, Writing – review & editing.
