# Supplementary material for: Effect of grazing and climatic factors on biodiversity-ecosystem functioning relationships in grassland ecosystems - a case study of typical steppe in Inner Mongolia, China
Source: Front Plant Sci. 2023 Dec 22;14:1297061. doi: 10.3389/fpls.2023.1297061 (PMC10770857; doi:10.3389/fpls.2023.1297061)
Supplement: Supplementary file 1 [file DataSheet_1.zip › Supplementary Material Presentation-Fig S1, Fig S2-Picture title and description.docx]

Figure S1

The changes of grassland plant community structure under grazing utilization and climate fluctuation from 2014 to 2022. (A) Proportion of ANPP in CK. (B) Proportion of ANPP in LG. (C) Proportion of ANPP in MG. (D) Proportion of ANPP in HG. CK represents control standard, LG represents light grazing, MG represents moderate grazing, and HG represents heavy grazing. PG represents perennial grasses, PF represents perennial forbs, SS represents shrubs and semi-shrubs, and AB represents annuals and biennials. ANPP represents aboveground net primary productivity.

Figure S2

The relative value of species diversity index from 2014 to 2022. The relative value of the species diversity index: the CK value was standardized to 0, and the corresponding other treatment index minus the CK diversity index value. (A), (B), (C) represent LG. (D), (E), (F) represent MG. (G), (H), (I) represent HG. CK represents control standard, LG represents light grazing, MG represents moderate grazing, and HG represents heavy grazing.
