# Supplementary figures and images for: Effect of grazing and climatic factors on biodiversity-ecosystem functioning relationships in grassland ecosystems - a case study of typical steppe in Inner Mongolia, China
Source: Front Plant Sci. 2023 Dec 22;14:1297061. doi: 10.3389/fpls.2023.1297061 (PMC10770857; doi:10.3389/fpls.2023.1297061)

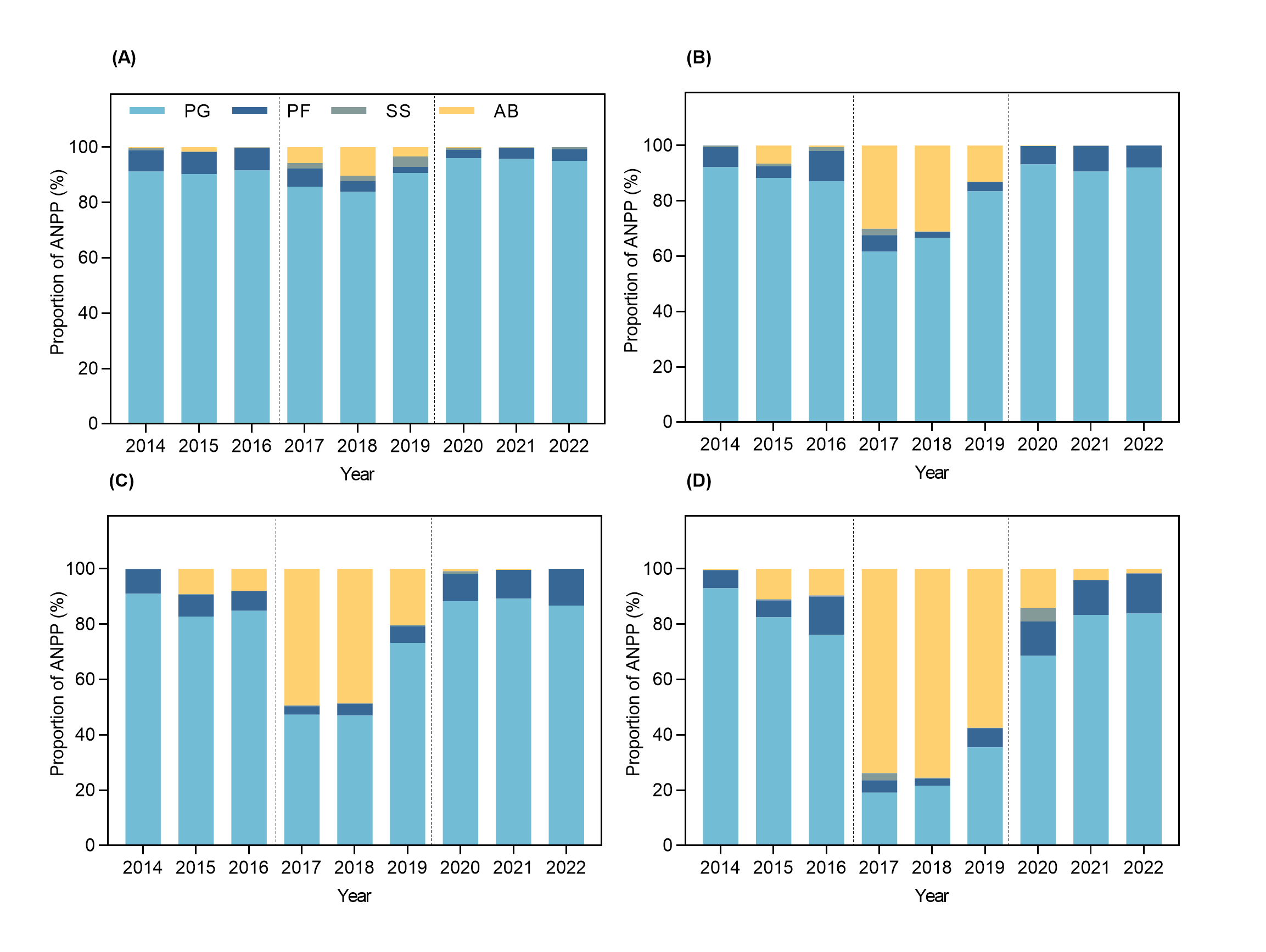

Supplement: Supplementary file 1 [file DataSheet_1.zip › Figure S1.tif]

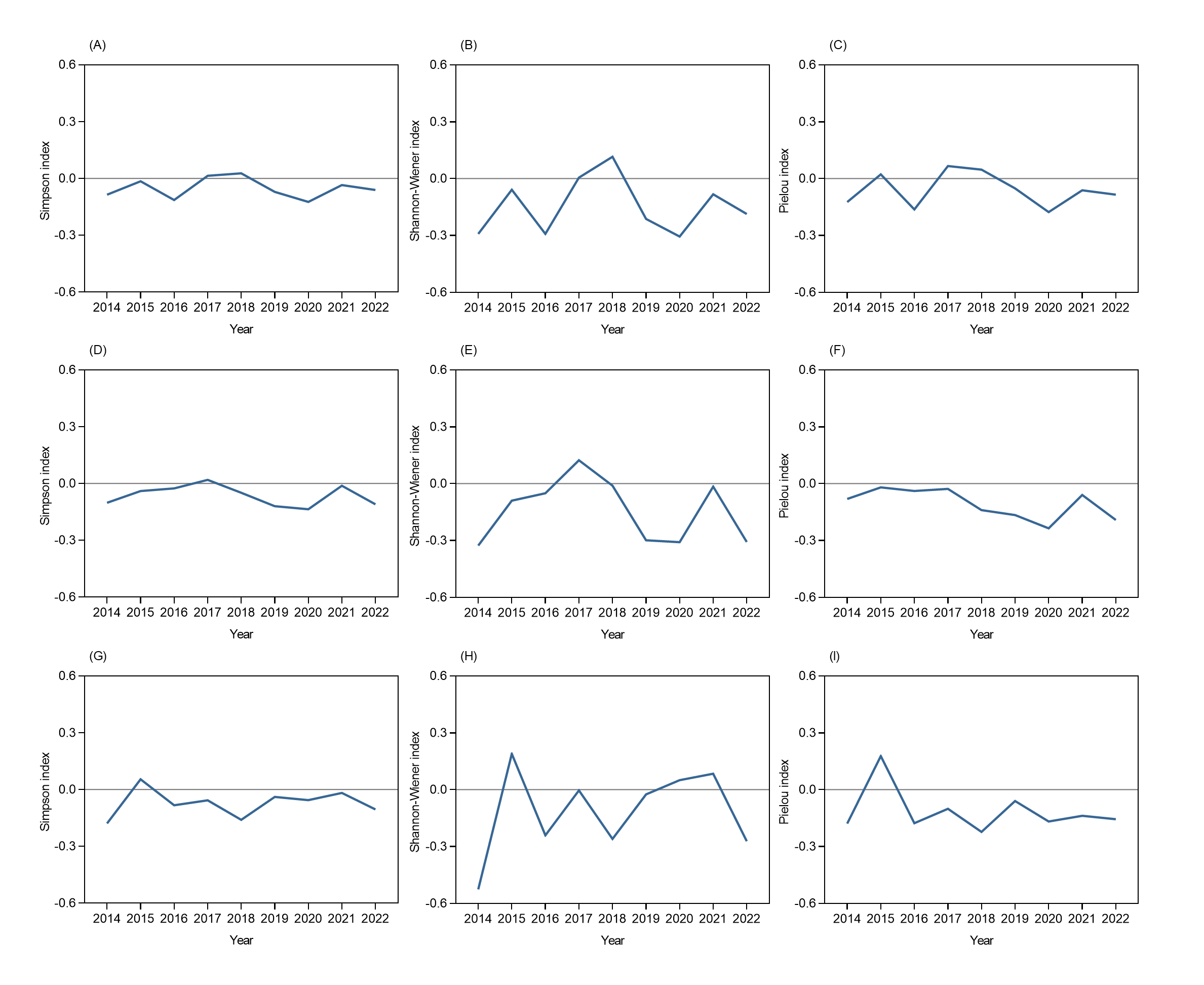

Supplement: Supplementary file 1 [file DataSheet_1.zip › Figure S2.tif]
